# Gender Differences in Associations Between Problematic Pornography Use and Symptoms of Depression and Anxiety: Insights From a Large‐Scale Study

**DOI:** 10.1155/da/5293861

**Published:** 2026-11-01

**Authors:** Ilaria De Luca, Giacomo Ciocca, Ornella Corazza, Gianluca Esposito, Luigi Tinella, Mónika Koós, Léna Nagy, Zsolt Demetrovics, Shane W. Kraus, Marc N. Potenza, International Sex Survey Consortium, Beáta Bőthe

**Affiliations:** ^1^ Department of Clinical Pharmaceutical and Biological Sciences University of Hertfordshire Hatfield UK herts.ac.uk; ^2^ Section of Sexual Psychopathology Department of Dynamic and Clinical Psychology, and Health Studies Sapienza University of Rome Rome Italy uniroma1.it; ^3^ Department of Psychology and Cognitive Science University of Trento Trento Italy unitn.it; ^4^ Department of Humanities Philosophy and Education University of Salerno Salerno Italy unisa.it; ^5^ Institute of Forensic Psychiatry and Sex Research Center for Translational Neuro-and Behavioral Science University of Duisburg-Essen Essen Germany uni-due.de; ^6^ Doctoral School of Psychology ELTE Eötvös Loránd University Budapest Hungary elte.hu; ^7^ Institute of Psychology ELTE Eötvös Loránd University Budapest Hungary elte.hu; ^8^ College of Human Sciences and Culture Flinders University Institute for Mental Health and Wellbeing Flinders University Bedford Park South Australia Australia flinders.edu.au; ^9^ Centre of Excellence in Responsible Gaming University of Gibraltar Gibraltar Gibraltar; ^10^ Department of Psychology University of Nevada Las Vegas Nevada USA unlv.edu; ^11^ Yale University School of Medicine New Haven Connecticut USA yale.edu; ^12^ Connecticut Council on Problem Gambling Wethersfield Connecticut USA; ^13^ Connecticut Mental Health Center New Haven Connecticut USA; ^14^ Département de Psychologie Université de Montréal Montréal Canada umontreal.ca; ^15^ Centre de Recherche Interdisciplinaire sur les Problèmes Conjugaux et les Agressions Sexuelles (CRIPCAS) Montréal Canada

**Keywords:** anxiety, compulsive-sexual behavior, depression, problematic-pornography use, psychopathology

## Abstract

**Introduction:**

Problematic pornography use (PPU) has been associated with symptoms of depression and anxiety. These associations have been studied in clinical and adolescent samples, but little attention has been paid to gender differences in international general population samples. This study examined relationships between PPU, anxiety symptoms, and depression symptoms, with a focus on potential gender differences.

**Method:**

Data were obtained from the International Sex Survey (ISS), a large‐scale, cross‐sectional study conducted across 42 countries. It involved 82,243 participants (Mean_age_ = 32.39 years, SD = 12.52; 39.6% men, 57.0% women, 3.4% gender diverse). Participants completed validated measures, including the Problematic Pornography Consumption Scale (PPCS) and the Brief Symptom Inventory’s (BSI‐18) depression and anxiety symptoms subscales. A one‐way ANOVA was used to assess gender differences, while regression analysis examined the role of gender (i.e., men, women, and gender‐diverse individuals) in the associations between psychological distress (i.e., depression and anxiety symptoms) and PPU.

**Results:**

Men reported the highest PPU levels, whereas gender‐diverse participants reported the highest anxiety and depression levels, followed by women. PPU was positively correlated with anxiety and depression symptoms, with small‐to‐moderate associations (*r* = 0.207 and *r* = 0.249, respectively). Gender significantly moderated both associations, with the steepest positive slopes among men, suggesting that they might rely more heavily on pornography as a maladaptive coping mechanism.

**Conclusions:**

This study highlights differences between PPU and mental health across gender identities. While gender‐diverse individuals experience the highest levels of psychological distress, men demonstrate the strongest link between such distress and PPU. These findings highlight the need for gender‐sensitive mental‐health interventions addressing PPU and its psychological implications.

## 1. Introduction

Sexuality is a fundamental aspect of many individuals’ lives and encompasses biologically rooted sexual motivation as well as behaviors shaped by personal, relational, and sociocultural factors. Sexual behaviors and desires vary substantially across individuals, and high‐frequency or nonnormative sexual behavior is not, by itself, evidence of disorder. Distinguishing nonproblematic from clinically significant sexual behavior therefore requires attention to impaired control, marked distress, and functional impairment, thereby reducing the risk of overpathologizing common or consensual behaviors [1–3]. Difficulties related to sexuality can nevertheless result in substantial distress [4]. Reflecting this distinction, the World Health Organization (WHO) included Compulsive Sexual Behavior Disorder (CSBD) in the 11th revision of the International Classification of Diseases (2018), where CSBD is described as an impulse control disorder characterized by a persistent pattern of failure to control intense, repetitive sexual impulses or urges resulting in repetitive sexual behavior. The pattern is manifested over an extended period of time (e.g., 6 months or more) and causes marked distress or significant impairment in important areas of functioning [5].

Up to ~80% of individuals with CSBD may experience problematic pornography use (PPU), which makes it the most clinically relevant manifestation of CSBD [6–9]. Pornography refers to professionally produced or consumer‐generated videos, still pictures, or other material intended to sexually arouse individuals [10]. Pornography currently is primarily consumed online, perhaps due to its perceived anonymity, affordability, and accessibility [11, 12]. Males who use the internet (47%) may be nearly twice as likely to watch pornographic content as females (27%), with the 18–27 age range being the most prevalent demographic group [13]. A survey of the US population showed similar figures, with 46% of men and 16% of women reporting they had viewed pornographic material in the previous week [14]. Recent literature reveals significant gender differences: compared to women, men typically consume more pornography in both time spent and frequency [15, 16] and tend to experience higher levels of PPU [17, 18]. Findings from the International Sex Survey (ISS) further corroborate these patterns, showing that men consistently report higher rates of PPU across different countries and sexual orientations, regardless of the assessment tool used [19]. Regarding sexual and gender minorities, recent studies suggest a more nuanced pattern [19]. Gender‐diverse individuals^1^ (e.g., nonbinary, genderfluid, and agender) tend to report lower levels of PPU (*M* = 32.06, SD = 18.54) than men (*M* = 38.55, SD = 19.85) but higher levels than women (*M* = 24.24, SD = 11.03). These findings underscore heterogeneity across gender identities and sexual orientations and suggest a more nuanced situation regarding gender differences.

Individuals experiencing PPU may use pornography to alleviate or eliminate stress or negative emotions [20]. Individuals using pornography more frequently have been found to have poorer mental health, in particular, symptoms of depression and anxiety [21, 22]. Depression is commonly characterized by persistent sadness, limited interests in or pleasure derived from daily activities, and emotional and physical symptoms that interfere with normal functioning, whereas anxiety often involves prolonged and excessive worry that significantly impairs managing everyday demands [23]. Some researchers suggest that findings lining PPU to affective concerns are influenced by reliance on clinical samples [24, 25] or samples with self‐identified PPU [26], while others focus on adolescents, who may be more prone to emotional fluctuations and dysregulation [27, 28].

Recent systematic reviews showed consistent associations between PPU and affective concerns, demonstrating a moderate association between PPU and depressive symptoms [13, 29–31]. Recent evidence also points to the roles of guilt, shame, and moral incongruence factors that may exacerbate both depressive feelings and PPU [18, 32], thereby highlighting a potentially complex relationship between PPU and depression. Minority stress, such as internalized stigma and discrimination, may intensify the link between PPU and depressive symptoms in cisgender sexual minority adults. For example, Lewczuk et al. [33] found internalized sexual stigma to be a significant predictor of PPU severity among gay and bisexual individuals. Additionally, in a large international sample, PPU was found to correlate with the severity of depression, anxiety, and stress, particularly among sexual minority students, where minority stress acts as a key mediator [34]. These data support a nuanced model in which both PPU and depressive symptoms are interconnected, especially under the influence of minority stress processes in LGBTQ+ (lesbian, gay, bisexual, transgender, queer/questioning, and other diverse sexual and gender identities) populations.

Anxiety has been positively correlated with CSBD, with both contributory and consequential roles implicated in its development and persistence [35]. Although variability in study designs and measurement tools precluded a metaanalytic calculation of a unified effect size, the authors emphasized a consistent positive association between anxiety and CSBD [35]. Individuals with generalized anxiety disorder (GAD), social anxiety disorder, and obsessive–compulsive disorder (OCD) may be more likely to exhibit compulsive sexual behaviors, often as a maladaptive coping mechanism to alleviate distress [3]. Moreover, young adults (18–35 years old) exhibit high prevalence estimates of CSBD. In this population, compulsive sexual behavior is positively associated with symptoms of anxiety, although specific effect sizes for these associations were not reported [36]. PPU, one of the most prevalent manifestations of CSBD, is also closely linked to anxiety [8, 37, 38]. While frequent pornography use itself is associated with higher anxiety levels, studies indicate that self‐perceived pornography addiction, the belief that one’s pornography consumption is excessive or uncontrollable, may be an even stronger predictor of anxiety than actual use frequency [39, 40]. Furthermore, recent findings indicate that PPU is significantly associated with heightened psychological distress, including elevated levels of social anxiety, suggesting that anxiety contributes to the clinical profile of individuals experiencing PPU [41, 42]. Sexual and gender minorities may also experience a stronger association between PPU and anxiety symptoms compared to heterosexual and cisgender individuals. For instance, Lewczuk et al. [33] reported that among cisgender sexual minority individuals, internalized sexual stigma and experiences of discrimination were positively associated with both PPU and symptoms of anxiety. Similarly, Jennings et al. [43] reported that minority stress contributes to the development and maintenance of compulsive sexual behaviors and cooccurring concerns, including anxiety, in LGBTQ+ populations.

In line with the self‐medication hypothesis [44, 45], pornography or other sexual behaviors may be used by some individuals to alleviate or suppress symptoms of anxiety and depression. However, the temporal ordering of these associations is not established. Psychological distress may precede and motivate pornography use, whereas pornography use, including use before a clinically problematic pattern becomes established, may also precede or contribute to later distress; common underlying factors may influence both [39, 42, 46]. Thus, the relationship may be bidirectional and may operate before the criteria for PPU are met. In addition, sexual and gender minorities, particularly transgender and gender‐diverse individuals, report higher levels of mental‐health concerns compared with cisgender individuals [47], especially anxiety and depression [48].

### 1.1. Knowledge Gaps, Study Aims, and Hypotheses

Despite growing evidence linking PPU to depression and anxiety, much of the existing literature relies on narrow or specialized samples, such as clinical populations [24, 25] or adolescents [27, 28]. This focus limits the generalizability of findings regarding the prevalence and correlates of PPU across gender identities, sexual orientations, and cultural contexts. For instance, most studies focus on cisgender men [49], potentially overlooking unique psychological and social factors that may promote pornography use in women, gender‐diverse individuals, and sexual minorities [12, 33]. Additionally, while some studies have documented elevated mental‐health risks in specific groups (e.g., college students, clinical samples, and sexual and gender minorities; [47, 48]), these findings are typically derived from smaller convenience samples and are not always replicated in cross‐national, large‐scale surveys. Consequently, there remains a gap in understanding how PPU and related mental‐health concerns, such as depression and anxiety, vary across diverse populations. This limitation restricts the generalizability of existing findings to broader populations, underscoring the need for additional research to inform public health interventions and clinical guidelines.

This study is the first to examine gender differences in the relationships between PPU, anxiety, and depression symptoms in a large, heterogeneous international community sample. The first objective was to determine whether PPU, depression, and anxiety symptom levels differed across gender groups (i.e., men, women, and gender‐diverse individuals). We hypothesized that PPU would be higher in men than in other gender groups, whereas depression and anxiety symptoms would be higher among women and gender‐diverse individuals than among men. The second objective was to examine the associations between anxiety symptoms, depressive symptoms, and PPU. We hypothesized that higher anxiety and depression symptom levels would be associated with higher PPU levels. Finally, we examined the potential moderating role of gender in these associations. We hypothesized that the associations of anxiety and depressive symptoms with PPU would be strongest among men, followed by gender‐diverse individuals and women. The study’s research question and hypotheses were preregistered on the OSF: https://osf.io/3urhy.

## 2. Methods

The ISS (http://internationalsexsurvey.org/) is an international, multilab, multilanguage study that used cross‐sectional and self‐report survey methods in 42 countries (preregistered study design: https://osf.io/uyfra/?view_only=6e4f96b748be42d99363d58e32d511b8)^2^. The survey was disseminated through a secure online platform (Qualtrics Research Suite) and was translated into 25 country‐specific languages from English. The time required to complete the survey ranged from 25 to 45 min. Before accessing the survey, all participants were required to provide written informed consent electronically via the Qualtrics platform; only individuals who provided consent were able to proceed with and complete the survey. Inclusion criteria were (a) being at least 18 years old (or the legal age to provide informed consent) and (b) understanding any of the languages in which the survey was available. Exclusion criteria were (a) failing two out of three attention questions [50] (Thomas and Clifford, 2017) and/or (b) producing contradictory response patterns. At the end of the survey, participants received a list of mental‐health services if they felt a need to consult a professional. As an incentive, we informed participants that we would donate 50 cents to nonprofit, sexuality‐related international organizations (e.g., World Association for Sexual Health) for every completed survey, with a maximum of a $1000 donation. The list of collaborating countries, detailed description of the translation and data collection procedures, and more details about the eligibility criteria are described in the study protocol [51]. For complete transparency of data use, all published papers and conference presentations are reported on the OSF pages (https://osf.io/uyfra/). The study was approved or deemed exempt by all collaborating countries’ national/institutional ethics review boards (https://osf.io/n3k2c/?view_only=838146f6027c4e6bb68371d9d14220b5) and was conducted in accordance with the Helsinki Declaration.

### 2.1. Participants

After data cleaning (detailed data cleaning procedure: https://doi.org/10.17605/OSF.IO/DK78R), a total of 82,243 participants (Mean age = 32.39 years, SD = 12.52, 39.6% men, 57.0% women, and 3.4% gender diverse) were included in the final dataset.

Participants’ detailed sociodemographic characteristics by country can be found at https://osf.io/n3k2c/?view_only=838146f6027c4e6bb68371d9d14220b5. For this study, all participants who completed the Problematic Pornography Consumption Scale (PPCS) and the Brief Symptom Inventory (BSI‐18) were included.

### 2.2. Measures

Participants were asked questions about their sociodemographic characteristics. For this study, we considered gender identity [52] as a variable of focus, while other sociodemographic characteristics were used to describe the sample. The participants were then asked specific questions about their use of pornography. The wording of each sociodemographic and sexuality‐related question and answer option and all scales in all languages can be seen at https://osf.io/jcz96/?view_only=9af0068dde81488db54638a01c8ae118.

The severity of the PPU in the past 6 months was assessed using the PPCS [53]. The 18‐item PPCS includes six factors: *salience* (three items, e.g., “I felt that pornography is an important part of my life.”), *tolerance* (three items, e.g., “I felt that I had to watch more and more pornography for satisfaction.”), *mood modification* (three items, e.g., “I used pornography to restore the tranquillity of my feelings.”), *conflict* (three items, e.g., “Watching pornography prevented me from bringing out the best in me.”), *withdrawal* (three items, e.g., “I became agitated when I was unable to watch pornography.”), and *relapse* (three items, e.g., “I unsuccessfully tried to reduce the amount of pornography I watch.”). Participants indicate their answers on a 7‐point scale (1 = “never”; 7 = “all the time”). Scoring 76 points or more indicates the risk of PPU [19, 53]

The study assessed the presence of depression and anxiety symptoms (BSI‐18) [54, 55] in the past 7 days. Participants had to indicate their answers on a 5‐point scale (0 = “not at all”; 4 = “extremely”). Scores ranged between 0 and 24 points for both scales.

### 2.3. Statistical Analysis

Analyses were performed using the software IBM SPSS Statistics (Version 29.0.2) [56] and Jamovi (Version 2.3.28) [57]. Prior to conducting the analyses, the assumptions of normality and homoscedasticity were examined. Although some variables did not follow a normal distribution, skewness and kurtosis values were within acceptable thresholds [58, 59], and the large sample size (*N* > 30) justified the use of parametric tests based on the central limit theorem.

To address the three study aims, we performed (a) group comparisons, (b) correlation analyses, and (c) moderation analyses. We conducted a one‐way ANOVA using Welch’s correction to examine differences in PPU, anxiety, and depression symptoms across gender groups (men, women, and gender‐diverse). Games–Howell posthoc tests were applied to assess pairwise group differences, given the unequal sample sizes and variance heterogeneity. To explore the overall associations between psychological distress and PPU, bivariate Pearson correlations were computed between PPU scores and both anxiety and depression symptoms. We then performed a moderation analysis to examine the moderating role of gender in the relationship between anxiety and depression symptoms (independent variables) and PPU (dependent variable). Gender was included in the moderation analysis, given the adequate sample sizes of gender groups, and was dummy‐coded to allow for comparison across the three groups. Anxiety and depression symptoms were included as predictors in two separate regression models to examine their relationship to PPU. Gender was included in the moderation analysis given the adequate sample sizes of gender groups. The *R*‐squared (*R* ^2^; e.g., higher values indicating better fit) was used to assess model fit, while the 95% confidence intervals were considered for the interpretation of the analysis of simple effects.

## 3. Results

### 3.1. Gender Differences in PPU, Depression Symptoms, and Anxiety Symptoms

The one‐way ANOVA revealed significant differences across gender groups for all three study variables. PPU scores showed a significant main effect of gender, *F*(6703) = 6805, ηp^2^ = 0.664, *p* < 0.001. Men reported the highest PPU scores (*M* = 38.55, SD = 19.85), followed by gender‐diverse individuals (*M* = 32.06, SD = 18.54) and women with the lowest levels (*M* = 24.24, SD = 11.03). Games–Howell posthoc tests indicated that every gender group differed significantly from the others (Table 1). The effect size was large, consistent with a strong influence of gender on PPU scores (Table 2).

**Table 1:** Games–Howell posthoc pairwise comparisons by gender.

Measure	Men/Women	Men/GD	Women/GD
	MD	MD	MD
PPU	14.3 ^∗∗∗^	6.5 ^∗∗∗^	−7.8 ^∗∗∗^
Anxiety	−2.15 ^∗∗∗^	−4.48 ^∗∗∗^	−2.33 ^∗∗∗^
Depression	−0.96 ^∗∗∗^	−4.56 ^∗∗∗^	−3.60 ^∗∗∗^

Abbreviation: GD, gender‐diverse, MD, mean difference, PPU, problematic pornography use.

^∗∗∗^ *p* < 0.001.

**Table 2:** Gender differences in problematic pornography use, depression and anxiety. One‐way ANOVA results.

Measure	Group	*N*	Mean (min–max)	SD	SE	ηp^2^
Problematic pornography use	Men	31,426	38.55 (18–126)	19.85	0.112	0.664
	Women	40,988	24.24 (18–126)	11.03	0.054	
	GD individuals	2609	32.06 (18–126)	18.54	0.363	

Anxiety	Men	30,051	5.27 (0–24)	5.13	0.029	0.336
	Women	43,377	7.42 (0–24)	5.76	0.027	
	GD individuals	2590	9.75 (0–24)	6.28	0.123	

Depression	Men	30,049	6.08 (0–24)	5.87	0.034	0.162
	Women	43,372	7.04 (0–24)	5.99	0.029	
	GD individuals	2589	10.64 (0–24)	6.77	0.133	

Abbreviation: GD, gender‐diverse, SD, standard deviation, SE, standard error, ηp^2^, partial eta squared.

Anxiety levels also differed significantly across gender groups, *F*(1766) = 6988, ηp^2^ = 0.336, *p* < 0.001. Women reported higher anxiety levels (*M* = 7.42, SD = 5.76) compared to men (*M* = 5.27, SD = 5.13), with gender‐diverse individuals having the highest anxiety levels (*M* = 9.75, SD = 6.28). These results align with the hypothesis that anxiety would be elevated in women and gender‐diverse individuals compared to that in men. The effect size was medium, reflecting a strong association between gender and anxiety symptoms (Table 2).

Depression scores varied significantly by gender, *F*(676) = 6976, ηp = 0.162, *p* < 0.001. Gender‐diverse individuals reported the highest depression scores (*M* = 10.64, SD = 6.77), followed by women (*M* = 7.04, SD = 5.99) and men with the lowest levels (*M* = 6.08, SD = 5.87). Posthoc analyses indicated significant differences between all groups, supporting the hypothesis that depression levels are higher among women and gender‐diverse individuals compared to men. The effect size was small, indicating gender‐based differences in depression symptoms (Table 2).

For both anxiety and depression symptoms, all posthoc comparisons remained significant at *p*  < 0.001, suggesting significant differences between all genders. Women and gender‐diverse participants reported higher symptom levels than men, and gender‐diverse participants differed significantly from women as well (Table 1). One‐way ANOVAs were performed on the complete records. Participants with missing data on any of the dependent variables or on gender were list‐wise deleted.

### 3.2. Associations Between Anxiety Symptoms, Depression Symptoms, and PPU

The correlation analysis revealed significant positive correlations between PPU scores and both anxiety and depression symptoms in the total sample. Specifically, the correlation between PPU and anxiety symptoms was *r* = 0.207, *p* < 0.001, and between PPU and depression symptoms *r* = 0.249, *p* < 0.001 (Table 3). According to Cohen’s [60] guidelines, these would be interpreted as small associations [60]. However, subsequent research has highlighted that such conventional thresholds may underestimate the practical significance of the psychological effects. Funder and Ozer [61] argued that correlations of approximately *r* = 0.20 should be considered medium‐sized effects as they often represent meaningful real‐world relationships. In line with these perspectives, the present correlations can be interpreted as small‐to‐moderate in magnitude. These findings support the hypothesis that more severe levels of anxiety and depression are associated with more severe PPU in the overall population. This step provides an estimate of the raw association between psychological distress and PPU across the entire sample before examining the potential moderating role of gender.

**Table 3:** Descriptive statistics and correlations between PPU, anxiety, and depression (*N* = 82,243).

Variable	*M*	SD	1	2	3
PPU	29.97	16.83	—	—	—
Anxiety	6.78	5.70	0.207 ^∗∗^	—	—
Depression	6.96	6.01	0.249 ^∗∗^	0.682 ^∗∗^	—

*Note*: All correlations are Pearson’s *r*.

Abbreviations: *M*, mean; PPU, problematic pornography use; SD, standard deviation.

^∗^ *p* < 0.05.

^∗∗^ *p* < 0.001.

### 3.3. Moderating Role of Gender in Associations Between PPU, Anxiety, and Depression

The moderation analysis suggested that gender significantly moderated the association between anxiety symptoms and PPU, *F*(2,69269) = 782, *p* < 0.001, and between depression symptoms and PPU, *F*(2,69263) = 674, *p* < 0.001. Specifically, men exhibited the highest slopes in the PPU‐anxiety and PPU‐depression symptom relationships. The association between anxiety symptoms and PPU was strongest among men (*B* = 1.142, 95% CI [1.108, 1.176], *β* = 0.379, *p* < 0.001), followed by women (*B* = 0.295, 95% CI [0.269, 0.322], *β* = 0.098, *p* < 0.001), and gender‐diverse individuals (*B* = 0.222, 95% CI [0.126, 0.317], *β* = 0.074, *p* < 0.001) (Table 4, Figure 1). Similarly, for depression symptoms, the association with PPU was strongest among men (*B* = 1.050, 95% CI [1.021, 1.080], *β* = 0.373, *p* < 0.001), followed by women (*B* = 0.341, 95% CI [0.316, 0.367], *β* = 0.121, *p* < 0.001), and gender‐diverse individuals (*B* = 0.310, 95% CI [0.222, 0.399], *β* = 0.110, *p* < 0.001) (Table 5, Figure 2). The models showed similar *R* ^2^ values for anxiety (0.221) and depression (0.229), respectively. These results suggest that men, relative to women and gender‐diverse individuals, experience stronger links between anxiety and depression symptoms and PPU.

**Figure 1:**
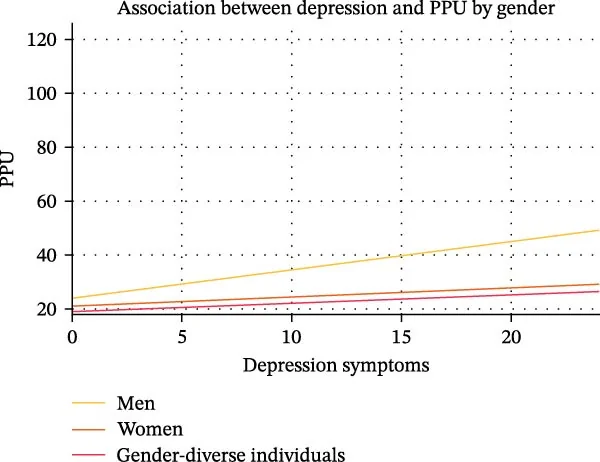
Moderation analysis of the role of gender in the association between depression symptoms (independent variable) and problematic pornography use (dependent variable).

**Figure 2:**
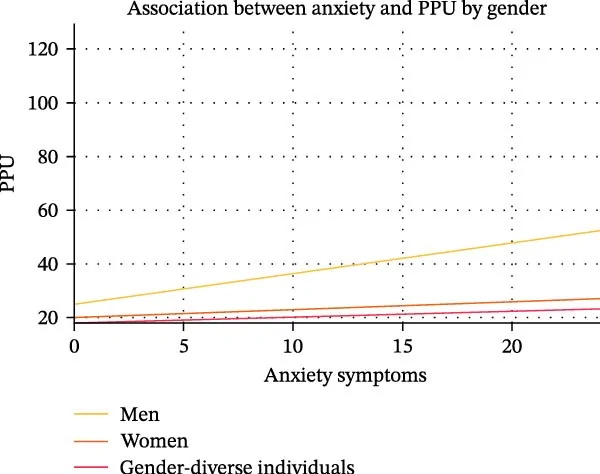
Moderation analysis of the role of gender in the association between anxiety symptoms (independent variable) and problematic pornography use (dependent variable).

**Table 4:** Moderation analysis of the role of gender in the association between depression symptoms (independent variable) and problematic pornography use (dependent variable).

Moderators levels	Estimate	SE	95% CI		*β*	*p*	*R^2^*
			LL	UL			
Gender							
Men	1.050	0.015	1.021	1.080	0.373	0.001	0.229
Women	0.341	0.013	0.316	0.367	0.121	0.001	—
GD individuals	0.310	0.045	0.222	0.399	0.110	0.001	—

Abbreviations: *β*, standardized coefficient; CI, confidence interval; GD, gender‐diverse; LL, lower limit; UL, upper limit.

**Table 5:** Moderation analysis of the role of gender in the association between anxiety symptoms (independent variable) and problematic pornography use (dependent variable).

Moderators levels	Estimate	SE	95% CI		*β*	*p*	*R^2^*
			LL	UL			
Gender							
Men	1.142	0.017	1.108	1.176	0.379	0.001	0.221
Women	0.295	0.013	0.269	0.322	0.098	0.001	—
GD individuals	0.222	0.048	0.126	0.317	0.074	0.001	—

Abbreviations: *β*, standardized coefficient; CI, confidence interval; GD, gender‐diverse; LL, lower limit; UL, upper limit.

## 4. Discussion

This study examined the associations between PPU, depression, and anxiety symptoms across different gender identities, with a specific focus on gender differences. While prior research has mostly focused on small and homogeneous samples without an emphasis on gender‐diverse populations [35, 49], this study addressed a key gap by using a large, diverse, cross‐national sample to explore how these associations manifest across genders. Understanding these differences is essential to developing tailored mental‐health interventions and informing public health strategies aimed at reducing PPU and psychological distress among different gender groups. The findings provide empirical support for the three proposed hypotheses, highlighting significant gender‐based variations in PPU and psychological distress, as well as the moderating role of gender in the associations between anxiety symptoms, depression symptoms, and PPU.

The first hypothesis proposed that men would exhibit higher levels of PPU, while depression and anxiety would be more severe among women and gender‐diverse individuals. The results supported this hypothesis, with men reporting significantly higher levels of PPU compared to those of women and gender‐diverse individuals. This finding is consistent with previous research indicating that men are more likely to engage in frequent pornography consumption and exhibit compulsive sexual behaviors [18]. More recent large‐scale and cross‐national studies have similarly found that men consistently report higher levels of PPU compared to other gender groups [8, 19, 37], further supporting the robustness of this gender difference across diverse populations. The higher prevalence of PPU in men may be explained by biological and sociocultural factors, including greater exposure to sexually explicit material and the normalization of pornography use as an expression of male sexuality [15]. Conversely, gender‐diverse individuals and women reported significantly higher levels of depression and anxiety symptoms compared to those of men. This aligns with the existing literature showing that gender‐diverse individuals are at increased risk for preexisting or cooccurring mental‐health issues due to experiences of minority stress, discrimination, and social exclusion [47, 48]. Similarly, women tend to exhibit higher levels of internalizing disorders, including depression and anxiety, which may be influenced by biological, social, and psychological factors, for example, hormonal fluctuations across the menstrual cycle [62], gendered socialization emphasizing emotional expressiveness [63], and greater exposure to interpersonal stressors such as caregiving burden or relational conflict [64, 65]. The elevated levels of anxiety/depression symptoms in these groups underscore the importance of considering the gender identity in mental‐health interventions.

The second hypothesis proposed that higher levels of anxiety and depression would be associated with more severe PPU. The results supported this hypothesis, revealing significant positive correlations between PPU and both anxiety and depression symptoms across the overall sample. These findings align with the self‐medication hypothesis [44], which suggests that individuals experiencing psychological distress may engage in maladaptive behaviors, such as excessive pornography consumption, to alleviate negative emotions [26]. Previous research has found that individuals with higher levels of anxiety and depression are more likely to use pornography as a coping mechanism [20]. However, while the associations between PPU and affective concerns were significant, the temporal directionality of this relationship remains unclear due to the cross‐sectional nature of the study. It is possible that individuals with preexisting anxiety and depression are more prone to PPU as a form of emotional regulation. Conversely, excessive pornography use may contribute to worsening mental‐health symptoms by reinforcing feelings of isolation, guilt, and diminished self‐esteem [66, 67]. Additionally, common factors (e.g., specific genetic or environmental factors) may predispose to the development of PPU and affective concerns.

The third hypothesis proposed that gender would moderate the associations between PPU and psychological distress. The results indicated that men exhibited higher slopes in the PPU‐anxiety and PPU‐depression symptom relationships compared to gender‐diverse individuals and women. A moderating role of male gender in these associations may be attributed to several factors. First, men reported the highest levels of PPU, suggesting potentially a stronger reliance on pornography use as a coping mechanism for emotional distress, in line with previous findings [51, 68]. This also aligns with prior research demonstrating that men are more likely to engage in externalizing behaviors to manage stress and negative emotions [46]. Additionally, sociocultural norms may contribute as men may be less likely to seek emotional support or utilize adaptive coping strategies, leading to greater engagement in PPU as an outlet for distress [69]. In contrast, while gender‐diverse individuals experienced the highest levels of affective concerns, their PPU levels were not as strongly associated with anxiety and depression symptoms. Speculatively, this may be explained by unique stressors faced by gender‐diverse populations, including discrimination, gender dysphoria, and limited social support [33]. Gender‐diverse individuals may engage in alternative coping mechanisms beyond pornography use, such as seeking online communities for validation or accessing mental‐health resources tailored to their needs. The relatively weaker effect among gender‐diverse individuals suggests that while they face significant psychological challenges, their use of pornography may not serve frequently as a primary mechanism for emotional regulation [42, 43]. Recent evidence further supports this interpretation. Pavanello Decaro et al. [70] highlight that mainstream pornography often objectifies and fetishizes gender‐diverse individuals, which may lead many of them to either avoid pornography altogether or engage with it in a critical and detached way. Such dynamics go beyond the mere absence of emotion‐regulation motives and may contribute to the weaker associations between pornography use and psychological distress observed among gender‐diverse participants in the present study.

The findings of this study have important diagnostic and clinical implications. First, clinicians should consider PPU not merely as a behavioral issue but as a potential correlate of underlying psychological distress, particularly symptoms of depression and anxiety [37, 39]. Routine mental‐health assessments should include questions about pornography use, especially in male clients, who may be less likely to disclose emotional difficulties but more likely to engage in avoidant or externalizing coping strategies such as excessive pornography consumption [46, 69]. For gender‐diverse individuals, assessments should also account for minority stress, discrimination, and gender dysphoria, which may exacerbate psychological distress [33, 71], even when not directly associated with elevated PPU levels.

From a clinical perspective, these findings underscore the need for gender‐informed therapeutic approaches. Mental health professionals should be trained to recognize how coping behaviors may differ by gender and to avoid pathologizing pornography use without contextual understanding [72]. Tailored interventions that address the unique psychosocial realities of each gender group are suggested by the findings. For men, treatment may involve promoting emotional literacy and developing alternative coping mechanisms [46], whereas for gender‐diverse individuals, care should be individualized and nondirective, exploring minority stress, gender‐related distress, cooccurring mental‐health concerns, the personal meaning and function of pornography use, and the individual’s treatment goals [47, 48, 71].

Intervention‐based research should explore the adaptation of evidence‐based treatments, such as cognitive–behavioral therapy (CBT), mindfulness‐based interventions, and acceptance and commitment therapy (ACT), which have shown promise in reducing CSBD and PPU and improving emotional regulation [3, 31, 46]. However, few studies have tested these modalities in the context of PPU specifically or among gender‐diverse populations, highlighting a gap and suggesting the importance of developing inclusive, empirically supported treatments.

### 4.1. Limitations and Future Directions

While this study provides novel insights into gender differences in the associations between PPU and symptoms of depression and anxiety, several study‐specific limitations should be acknowledged.

First, the cross‐sectional design prevents causal inferences about the direction of the association between PPU and psychological distress. Anxiety or depression may precede and contribute to PPU, pornography use, including use before a problematic pattern is established, may precede or contribute to later distress, and shared vulnerabilities may influence both. Longitudinal or experimental designs are needed to test these competing temporal pathways.

Second, although gender‐diverse individuals were included in the analyses, their proportion in the total sample was relatively small compared to that of men and women. This may have limited the statistical power to detect more nuanced effects within this group or to capture the full heterogeneity of gender‐diverse experiences. The ISS used advertisement‐based community recruitment rather than probability sampling, and the achieved analytic sample contained fewer men (39.6%) than women (57.0%), despite the protocol’s aim for an approximately balanced recruitment of men and women [51]. The sample should therefore not be considered population representative, and gender‐group proportions should not be interpreted as prevalence estimates.

Third, the use of self‐report measures, while efficient for large‐scale data collection, may have introduced reporting biases, such as social desirability or underreporting of sensitive behaviors.

Fourth, the data did not include contextual or qualitative information regarding pornography use (e.g., type of content consumed, motivations, or moral incongruence), which may further shape the relationship between PPU and affective symptoms.

Finally, although the ISS provides a rich cross‐national dataset, the present analyses merged participants across countries and did not model cross‐cultural variation. Participating countries differ in dominant religious traditions, norms surrounding sexuality, access to and regulation of pornography, and social and legal contexts for gender diversity. These factors may influence pornography use itself, moral incongruence or distress related to use, willingness to disclose pornography consumption, and the willingness or opportunity to self‐identify as gender‐diverse [18, 32, 66].

Together, these limitations highlight important directions for future research, particularly the need for longitudinal and culturally sensitive investigations examining the causal pathways between PPU and psychological distress across diverse gender identities. General limitations related to the ISS are discussed further on the study’s OSF page (https://osf.io/n3k2c/files/6kscb?view_only=83814%206f6027c4e6bb68371d9d14220b5).

## 5. Conclusions

The study findings suggest nuanced and clinically significant relationships between PPU and depression and anxiety symptoms. Crucially, the results reveal that these associations are not uniform across gender groups: while gender‐diverse individuals reported the highest overall levels of psychological distress, it was men who showed the strongest association between depression and anxiety symptoms and PPU levels. This suggests that pornography use may serve as a maladaptive coping strategy, particularly among men, with important implications for both diagnosis and treatment. These findings highlight the need for mental‐health professionals to systematically assess for PPU in the context of emotional distress and to consider gender‐specific pathways in how individuals may regulate negative affect. Clinically, this calls for the development and rigorous testing of gender‐informed treatment approaches, such as emotion‐regulation‐focused modules for men and minority‐stress‐informed interventions for gender‐diverse individuals, whose explicit goal is to reduce PPU and alleviate potential cooccurring symptoms of depression and anxiety.

## Author Contributions

Conceptualization: Ilaria De Luca, Beáta Bőthe, Giacomo Ciocca, and Ornella Corazza. Methodology, formal analysis: Ilaria De Luca, Beáta Bőthe, Giacomo Ciocca, Ornella Corazza, Gianluca Esposito, and Luigi Tinella. Data curation: Beáta Bőthe, Léna Nagy, Mónika Koós, Zsolt Demetrovics, Shane W. Kraus, Marc N. Potenza, Giacomo Ciocca, Ornella Corazza, and ISS Consortium. Writing – original draft: Ilaria De Luca. Writing – review and editing: Beáta Bőthe, Léna Nagy, Mónika Koós, Zsolt Demetrovics, Shane W. Kraus, Marc N. Potenza, Giacomo Ciocca, Ornella Corazza, Gianluca Esposito, Luigi Tinella, and ISS Consortium. Supervision: Beáta Bőthe, Léna Nagy, Mónika Koós, Zsolt Demetrovics, Shane W. Kraus, Marc N. Potenza, Giacomo Ciocca, Ornella Corazza, Gianluca Esposito, and Luigi Tinella. Project administration: Beáta Bőthe, Léna Nagy, Mónika Koós, Zsolt Demetrovics, Shane W. Kraus, Marc N. Potenza, Giacomo Ciocca, and Ornella Corazza.

## Funding

This study used data from the International Sex Survey. Individual members of the International Sex Survey Consortium received funding from various institutions and funding bodies, as reported in the Conflicts of Interest section.

## Disclosure

The authors attest that the information provided in the Conflicts of Interest section has had no influence on the design of this study or its outcomes. The funding sources had no role in the design of the study, data analysis, interpretation of results, or preparation of the manuscript.

## Ethics Statement

The study was conducted in accordance with the Declaration of Helsinki. Ethical approval for the International Sex Survey was obtained or deemed exempt by the institutional or national ethics review boards of all participating countries. All participants provided informed consent electronically before accessing the survey. Further details regarding ethical approvals are available on the International Sex Survey OSF project page.

## Conflicts of Interest

**Main Authors:** Shane W. Kraus discloses that he has received funding from the International Center for Responsible Gaming, M.G.M Resorts International, Center for the Application of Substance Abuse Technologies, Taylor Francis, Springer Nature, The Nevada Problem Gambling Project, Sports Betting Alliance, and Kindbridge Research Institute. Marc N. Potenza discloses that he has consulted for and advised Baria‐Tek and Boehringer Ingelheim; been involved in a patent application with Yale University and Novartis; received research support from the Mohegan Sun Casino and the Connecticut Council on Problem Gambling; consulted for or advised legal, nonprofit, healthcare, and gambling entities on issues related to impulse control, internet use, and addictive behaviors; performed grant reviews; edited journals/journal sections; given academic lectures in grand rounds, CME events, and other clinical/scientific venues; and generated books or chapters for publishers of mental‐health texts. **ISS Consortium:** Sophie Bergeron was supported by a Tier 1 Canada Research Chair;Julius Burkauskas received support from the WUN Research Development Fund (RDF) 2021; Jesús Castro‐Calvo received support from the Department of Education, Culture, Univiersities, and Employment, Valencian Government (Grant CIGE/2024/063);Lijun Chen was supported by the National Social Science Foundation of China (Grant 19BSH117); Rita Csako was supported by the Auckland University of Technology, 2021 Faculty Research Development Fund; Hironobu Fujiwara was supported by Grant‐in‐Aid for Transformative Research Areas (A) (Japan Society for The Promotion of Science, Grant JP21H05173), Grant‐in‐Aid for Scientific Research (B) (Japan Society for The Promotion of Science, Grant 21H02849), Grant‐in‐Aid for Scientific Research (C) (Japan Society for The Promotion of Science, Grant 23K07013), The Telecommunications Advancement Foundation, and The Smoking Research Foundation; Roman Gabrhelík was supported by Charles University Institutional Support Programme Cooperatio‐Health Sciences; Mateusz Gola was supported by the National Science Centre (Grant 2021/40/Q/HS6/00219); Joshua B Grubbs was supported by grants from the Kindbridge Research Institute, the International Center for Responsible Gaming, and the Problem Gambling Network of Ohio; Karol Lewczuk was supported by Sonatina grant awarded by National Science Centre, Poland, Grant 2020/36/C/HS6/00005; Chung‐Ying Lin was supported by the WUN Research Development Fund (RDF) 2021 and the Higher Education Sprout Project, the Ministry of Education at the Headquarters of University Advancement at the National Cheng Kung University (NCKU); Christine Lochner received support from the WUN Research Development Fund (RDF) 2021; KL was supported by Charles University Institutional Support Programme Cooperatio‐Health Sciences; Percy Mayta‐Tristán was supported by Universidad Cientifica del Sur; Gábor Orosz was supported by the ANR grant of the Chaire Professeur Junio; Gabriel C Quintero Garzola was supported by the SNI Number 073–2022 (SENACYT, Rep. of Panama); Kévin Rigaud was supported by a funding from the Hauts‐de‐France region (France) called “Dialogue Stratégique de Gestion 2 (DSG2).”

## Endnotes

1

2

## Data Availability

The data that support the findings of this study are available from the corresponding author upon reasonable request.

## Footnotes

1
^1^The term “gender‐diverse” refers to individuals whose experience of gender does not conform to the traditional male–female binary, including those who identify with multiple genders (bigender), no gender (agender), or whose gender fluctuates over time (genderfluid) [73].

2
^2^Egypt, Iran, Pakistan, and Romania were included in the study protocol paper as collaborating countries [51]; however, it was not possible to get ethical approval for the study in a timely manner in these countries. Chile was not included in the study protocol paper as a collaborating country [51] as it joined the study after the publication of the study protocol. Therefore, instead of the planned 45 countries [51], only 42 individual countries are considered in the present study; see details at https://osf.io/n3k2c/.
